# Supplementary material for: Loss of Aryl Hydrocarbon Receptor Favors K-RasG12D-Driven Non-Small Cell Lung Cancer
Source: Cancers (Basel). 2021 Aug 13;13(16):4071. doi: 10.3390/cancers13164071 (PMC8394265; doi:10.3390/cancers13164071)
Supplement: Supplementary file 1 [file cancers-13-04071-s001.zip › cancers-1340674-supplementary.pdf]

Supplementary Figure S1

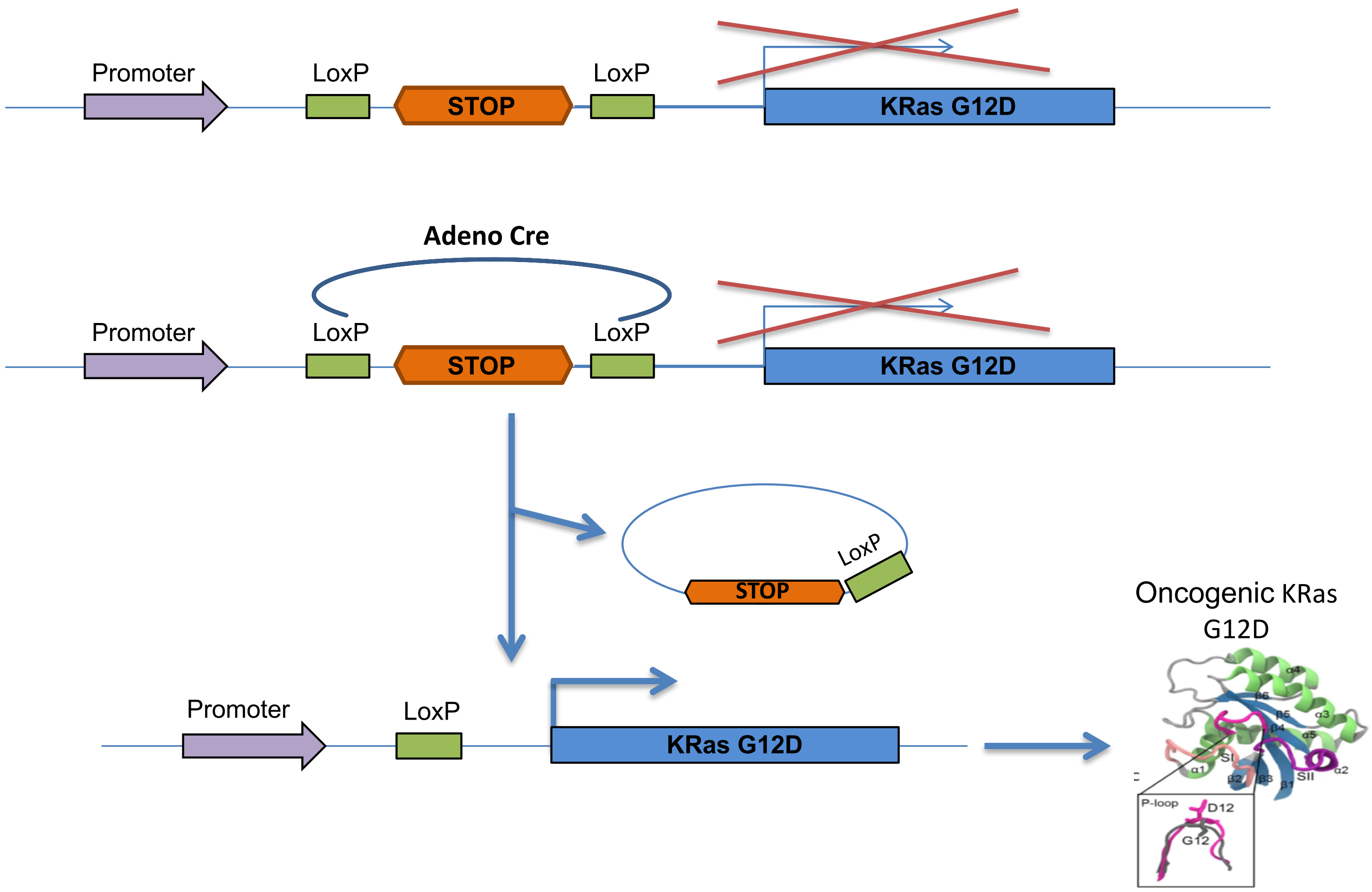

Supplementary Figure S2

(A) Ahr mRNA expression from RNA-seq analysis of tumor and non-tumor samples.
(B) Ahr mRNA expression in organoids from $Kras^{G12D/+};Ahr^{+/+}$ mice.

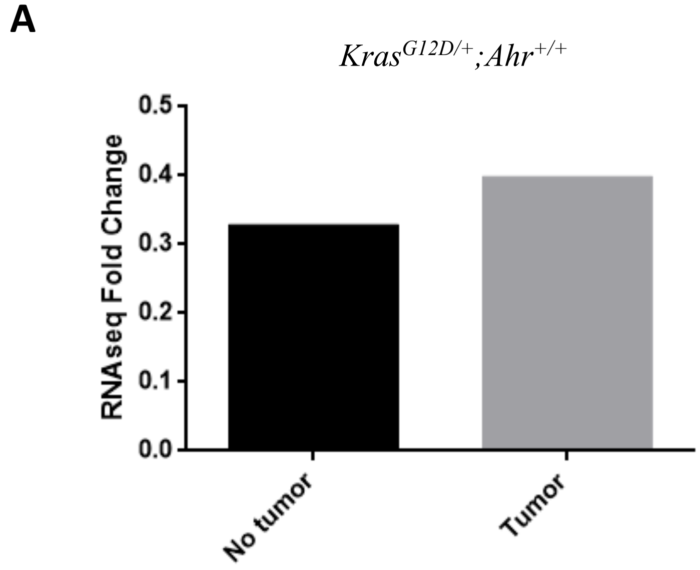

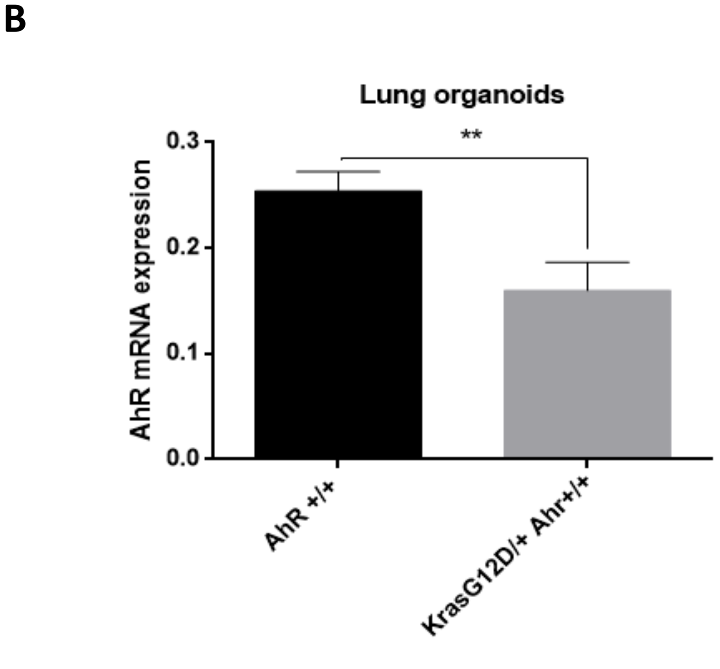

Supplementary Table S1

Oligonucleotide sequences used for mRNA expression analysis

Gene name	Sequence 5´- 3´
AhR	Fw: AGCCGGTGCAGAAAACAGTAA<br>Rv: AGGCGGTCTAACTCTGTGTGT
KRas^{G12D}	3´universal: AAAGTCGCTCTGAGTTGTTAT<br>5´mutant: GGAGCGGGAGAAATGGATATG<br>5´wild type: GCGAAGAGTTTGTCCTCAACC
Oct4	Fw: AGAGGGAACCTCCTCTGAGC<br>Rv: CCAAGGTGATCCTCTTCTGC
Nanog	Fw: CAAGGGTCTGCTACTGAGATGCTCTG<br>Rv: TTTTGTTTGGGACTGGTAGAAGAATCAG
Sox2	Fw: CGTAAGATGGCCCAGGAGAA<br>Rv: GCTTCTCGGTCTCGGACAAA
Klf4	Fw: TTTCTCGCCTGTGTGAGTTC<br>Rv: CACAAGTCCCCTCTCTCCAT
c-Myc	Fw: CCTGACGACGAGACCTTCA<br>Rv: TGGTAGGAGGCCAGCTTCT
Gapdh	Fw: TGAAGCAGGCATCTCAGGG<br>Rv: CGAAGGTGCAAGAGTGGGAG
